# Avoiding hERG-liability in drug design via synergetic combinations of different (Q)SAR methodologies and data sources: a case study in an industrial setting

**DOI:** 10.1186/s13321-019-0334-y

**Published:** 2019-02-02

**Authors:** Thierry Hanser, Fabian P. Steinmetz, Jeffrey Plante, Friedrich Rippmann, Mireille Krier

**Affiliations:** 1 Lhasa Limited, Leeds, UK; 2 Merck KGaA, Darmstadt, Germany

**Keywords:** hERG, QSAR, Machine learning, Public–private data sharing, Expert system, SOHN, Combining models, Temporal study

## Abstract

In this paper, we explore the impact of combining different in silico prediction approaches and data sources on the predictive performance of the resulting system. We use inhibition of the hERG ion channel target as the endpoint for this study as it constitutes a key safety concern in drug development and a potential cause of attrition. We will show that combining data sources can improve the relevance of the training set in regard of the target chemical space, leading to improved performance. Similarly we will demonstrate that combining multiple statistical models together, and with expert systems, can lead to positive synergistic effects when taking into account the confidence in the predictions of the merged systems. The best combinations analyzed display a good hERG predictivity. Finally, this work demonstrates the suitability of the SOHN methodology for building models in the context of receptor based endpoints like hERG inhibition when using the appropriate pharmacophoric descriptors.

**Electronic supplementary material:**

The online version of this article (10.1186/s13321-019-0334-y) contains supplementary material, which is available to authorized users.

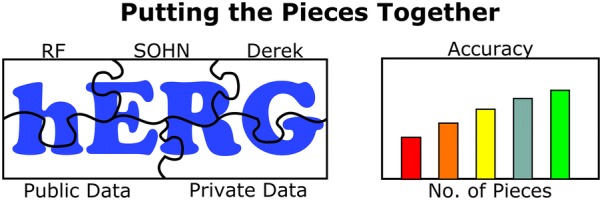

## Introduction

The inhibition of the human ether-a-go-go (hERG) ion channel may cause QT interval prolongation, which eventually can result in torsades de pointes (TdP) [1] and even death. Hence cardiotoxicity caused by the inhibition of hERG is a major liability within the drug development process. To avoid such a severe adverse effect, it makes good sense to screen all potential drug candidates for risk against blocking the hERG channel. It is therefore highly desirable to have a good means of predicting hERG activity, and for this purpose in silico systems provide a low cost solution that can be applied to the large datasets in early drug discovery. As such, a large number of different models have been developed. Herein, we present a new model, and a thorough comparison of its results using both public training data as well as mixing it with privately held data to tune the results towards a specific area of chemical space. We also examine the combination of different models, including both a traditional QSAR random forest (RF) model and an expert rule-based system along with the newly introduced self-organising hypothesis networks (SOHN) model.

**Fig. 1:**
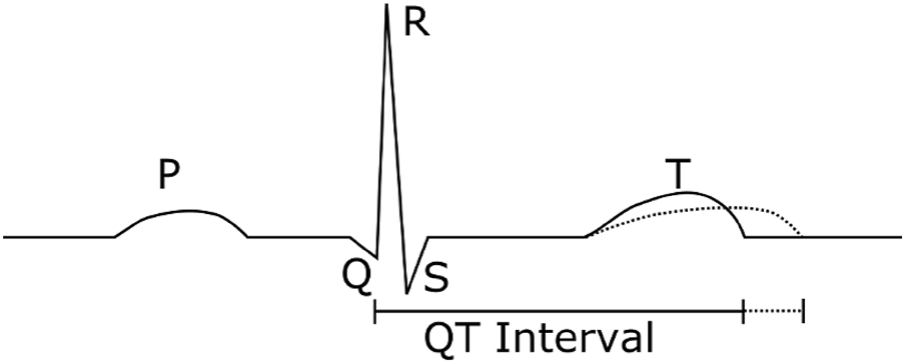
Idealised illustration of a QT interval prolongation as measured by electrocardiography

The hERG channel is a transmembrane ion channel to allow the passage of potassium ions through the cellular membrane and is related [2] to the ether a go-go (eag) gene discovered in fruit flies. It is named after the shaking behaviour observed in drosophila exposed to ether vapour [3] that was reminiscent of dancers at the Whisky a go-go bar in California. Inhibition of the hERG potassium channel has been shown to induce long-QT syndrome [4] by inhibiting the repolarisation of cardiac cells. By blocking the efflux of potassium ions, this lengthens the amount of time it takes to regenerate the cardiac action potential causing changes to the refractory period post-contraction (Fig. 1). This lengthening in time can cause TdP [1], a polymorphic ventricular tachycardia, which can ultimately cause syncope and rarely can cause cardiac failure and death. This is an unacceptable risk for most pharmaceuticals, and therefore a number have been withdrawn from use due to their ability to inhibit the hERG channel, such as cisapride [5] and terfenadine [6].

It is therefore advantageous, in pharmaceutical development, to screen for hERG liability early in development to avoid the expense of investigating compounds that will ultimately fail the more stringent risk assessments of ICH S7B or tests in accordance with FDA guidance E14. A number of different in vitro assays have been developed to screen for hERG inhibition, including a fluorescence assay, radioligand binding, and electrophysiology measurements using a patch-clamp assay [7], which is the current gold standard. The inhibition of the hERG channel appears to follow a defined pharmacophore [8, 9] with a number of rules of thumb for medicinal chemists to follow. In addition, a number of QSAR studies have been investigated using various descriptors, from simple logP and TPSA analysis to more complicated 3D calculated quantum parameters. However, the performance of different models is difficult to compare without a benchmarking test set. There are also differences in the activity levels for dichotomising a compound to active or inactive, with threshold IC_50_ levels set anywhere from 1 to 40 μM. With such a large range some compounds can shift from active to inactive in different studies, as such we are only able to take the authors recommendations for their own performance. There are two main classes of prediction of hERG activity: those trying to predict a binarised system or those trying to predict the continuum of activity of the compounds towards blocking the hERG channel itself. The binarised systems condense each compound down to a single active or inactive call, usually using a threshold value to define activity. The continuous systems instead use a regression method to try and predict the full activity over a range. To simplify the performance analysis a number of the continuous methods end up dichotomising the results after prediction, again using a threshold value. Across both methods a multitude of different modelling techniques have been applied. Wang et al. [10] have used recursive partitioning on a moderate (587 compounds) sized dataset to find pharmacophores. They then used either naive Bayes classifier or support vector machines to integrate multiple pharmacophores into a single call, using active as below the threshold value of 40 μM. Schyman et al. [11] used a similarity searching approach to classify hERG blockers, training with the same dataset as Wang, but with a different threshold value of 10 μM. They used both 2D and 3D similarity approaches along with a conservative call approach where if either 2D or 3D were deemed active then the overall call was active. Their method gives an overall Cohen’s kappa of 0.56 for the combined model. Barakat et al. [12] took the classification model to the extreme by building a homologous model to the hERG channel with a long molecular dynamics simulation, followed by docking different compounds, each consisting of multiple low energy poses, into the channel and examining the interaction energy for strength of binding. They were able to correctly identify 9 out of 10 hERG active drugs, but the time required for their method makes it impossible to use in a routine manner. The most thorough analysis of public data was done by Czodrowski [13], where he took the available IC_50_ and K_i_ values in ChEMBL [14] and used a random forest to examine the predictivity. He found a Matthews correlation coefficient (MCC) of 0.4 and the best simple accuracy reported was 90%, using two different threshold values of 1 and 10 μM. Li et al. [15] used Czodrowski’s dataset but applied multiple different descriptors and machine learning techniques to find a consensus model with a MCC of 0.62. Su et al. [16] have used a combination of traditional QSAR descriptors, VolSurf-like descriptors and 4D fingerprints [17] to predict the pIC_50_ of hERG inactivation using partial least squares. They then binarised their prediction for analysis using a cutoff value of 40.7 μM and found a simple accuracy measure of up to 90%. Gavaghan et al. [18] also used a system of different descriptors along with different PLS models to generate a hERG model, which has shown a RMSE of 0.5 against temporally shifted data at AstraZeneca. Passini et al. [19] have developed a series of in silico Ion channel models that are able to predict TdP risk with greater accuracy than animal models. Munawar et al. [20] have developed a pharmacophore model by extracting important interaction sites from known hERG active drugs in three dimensional space that had a MCC performance of 0.72 with a small test set consisting also of known hERG active drugs. Chemi et al. [21] have also used a similar 3D pharmacophore approach, but with a regression model to predict the inhibition constant of the hERG channel with good accuracy.

The currently described work does not intend to outperform any previously mentioned models as a primary objective. Instead we aim to explore the possible synergistic effects between existing in silico prediction systems as well as between different data sources and finally the combination of all these elements. To accomplish this study, we will involve 3 model types and 2 data sources; public and private.

For the public data source we used ChEMBL (version 23) [14], provided by the European Bioinformatics Institute. It offers a searchable database of both compounds and targets with their associated bioactivities. These bioactivities are drawn from the literature across a wide variety of different assays. Our way of working with the information is to condense it down into a single binary call. This allows us to compare the results of different bioactivity types to each other by applying a simple set of threshold values, in an analogous manner to what was described in earlier papers [22, 23]. After this condensation we have a single binary call for each compound which gave us a dataset suitable for modelling. This dataset, although quite large at 7681 compounds, is still unable to completely cover interesting chemical space. Another database has been developed [24] containing 291,219 compounds, but this work was complete before the publication of that database. Pharmaceutical companies tend to exploit narrow areas of chemical space. Thus it is helpful to have training data direct from these companies. This allows us to learn their focused area of chemical space, as well as use the large area of chemical space covered by ChEMBL to cover compounds at the periphery of their space. This private data also tends to be of a higher quality [25], simply due to the fact that it is gathered according to a single set protocol, usually within a single lab, meaning that the results are more consistent. For the private data, we will use in-house data from Merck as part of a data sharing initiative.

In addition to combining public and private data, we aim to combine three different models, two statistical and one expert system, to try to obtain the best performance. The statistical methods are a random forest (RF) and a self-organising hypothesis network [26] (SOHN), and the expert system is Derek Nexus [27] (version 5.0.2) developed by Lhasa Limited. Derek Nexus consists of a number of structural alerts that have been designed by an expert toxicologist. These alerts consist of manually compiled 2D structural patterns that attempt to capture the known hERG pharmacophores in a systematic manner. These different systems all have their own individual strengths and therefore the sum of the systems should have better performance than any individual system.

To explore the possible combinations of data sources and in silico models, we set up a series of 5 experiments covering 11 different models. To augment the challenge and simulate a realistic prospective prediction context, the performance of each model will be evaluated against a time split training and test sets [28]. In fact, this testing method is made even more challenging for the models as in silico assessment was used to direct the selection of compounds to synthesise, thereby prioritising compounds that existing models predicted as hERG inactive and leading to a negatively biased test set. This also means that known positives are, to some extent absent, from the time-split test set as the synthesis of new compounds is guided away from any hERG actives; the remaining positive structures usually exhibit unexpected structural triggers for hERG activity and require well trained models.

## Methods

### Modelling methodologies

#### Derek

Derek Nexus is an expert system which uses structural alerts to identify known toxicophores in a compound. In Derek Nexus (version 5.0.2), there are five alerts for the hERG endpoint. These alerts were built using publicly available data, as well as data that has been donated from Lhasa members. Each alert was developed by a Lhasa scientist looking at the structure activity relationship (SAR) of hERG inhibition across a multitude of structures, distilling the data down into patterns that capture the activity of the molecules.

#### Self-organising hypothesis network

The self-organising hypothesis network model (SOHN) is based on methodologies developed by Lhasa Limited [26]. This model takes a labelled dataset and learns the activity based on features present in the molecule. The feature used for predicting hERG consists of atom pairs where each atom of the pair is also typed with a typing system developed by Lhasa Limited [29], which is extended from the Ghose and Crippen atom types [30]. In essence, the molecule is represented by all possible atom pairs of between 2 and 20 topological bond distance. Each atom of the pair is further refined to not just its element, but also considering the local environment thereby capturing further information about the hybridisation as well as other atoms directly bonded. The presence of an atom pair is a potential toxicophoric hypothesis. The set of most relevant hypotheses is selected using recursive partitioning then organised into a hierarchical hypothesis network and used to generate a prediction. Each hypothesis in the network is supported by a set of examples from the training set and can be used as a local *k* nearest neighbors (kNN) model based on these supporting compounds. The most specific hERG hypotheses applicable to the query compound are identified and their corresponding kNN predictions are combined into a final call. For each prediction there is a confidence value associated, which is based on the similarity of the query compound to the kNN for each hypothesis, and the distribution of their hERG labels. The confidence value ranges from 0 to 1, where 1 is either an exact match to the training set, or a full agreement between the labels of the nearest neighbours.

#### Random forest

The random forest model (RF) was implemented in Scikit-learn 0.17 in python 2.7.11. Hyperparameter and feature selection were based on previous studies at Merck to optimise prior performance. The descriptors used were a set of physio-chemical properties, calculated by RDKit [31] (see Additional file 1) as well as Morgan fingerprints of radius 4 with a 4 k width and Feature Morgan fingerprints using again a radius of 4 and 4k width. For each prediction, there is also a confidence score (CS), which is the arithmetic mean of the maximum similarity to the training data and the prediction probability of the machine learning algorithm. The value scales from 0 to 1 but usually lies between 0.5 and 1 with 1 being 100% confident that the prediction is correct.

1 $${\text{CS}} = \frac{{{\text{Max}}\left( {{\text{similarity}}\;{\text{to}}\;{\text{training}}\;{\text{data}}} \right) + {\text{model}}\;{\text{probability}}}}{2}$$

### Data curation

The publicly available data was gathered from ChEMBL [14] by searching for compounds (7861) with bioactivities against the hERG channel. These bioactivities were binarised into an active/inactive call by either taking the authors call, or applying a threshold value to a numeric score. These threshold values are different depending on the bioactivity type (i.e. IC_50_, EC_50_, K_a_ etc.) but all are an attempt to establish a cutoff value equivalent to an IC_50_ of 10 μM. When a compound had multiple bioactivities they were merged into a single call. A conservative method was used where a single active was sufficient to result in an active overall call. The resultant dataset is close to balanced being 46.7% active and 53.3% inactive.

The Merck private dataset was obtained experimentally using Patchliner™, which is an automated whole cell patch clamp assay using HEK293 cells expressing Kv11.1. The procedure is similar to the assay described by Polonchuk [32], except that the experiment is performed at room temperature. The standard screen was performed using compounds dosed in 0.1% DMSO and tested as a single concentration (10 μM). Any compound eliciting over 50% inhibition at that concentration was classified as active and dose response curve is then measured in order to determine the IC50 value. If duplicate values were present the mean value of the inhibition was calculated and then binarised to active/inactive using the 50% threshold at 10 μM. To simulate a real case in industry, this dataset was split into a training set and a test set using a temporal split. The training set of 7515 compounds consisted of all data from the 25th of March 2010 to the 13th of April 2017. The time-split test set of 316 compounds consisted of the results from the 20th of April 2017 to the 23rd of June 2017. Compounds are unique to each dataset, making it impossible to have a compound in both the training and test set.

### Evaluation

A comprehensive set of statistical metrics were used to evaluate of the efficacy of the different models. These metrics, which are based on the typical confusion matrix (Additional file 1), were accuracy (ACC), sensitivity (SENS) (also known as recall or true positive rate), specificity (SPEC) (also known as true negative rate), positive predictive value (PPV) (also known as precision) and negative predictive value (NPV) [33, 34]. As the test set was significantly unbalanced, we also calculated balanced accuracy (BA) [35], Matthews correlation coefficient (MCC) [36] and Cohen’s kappa coefficient (KAPPA) [37] due to their capability of dealing with unbalanced data. All equations adapted for binary classification models are detailed in the Additional file 1.

## Results and discussion

All the statistical models and the Derek expert system were evaluated against the test set. This validation process is challenging from chemical space perspective and provides a good insight on the potential of delivering accurate hERG predictions in the context of newly developed compounds. The validation is made even more difficult due to the strong negative bias of the dataset (approximatively 75% negatives vs 25% positives). Such bias is typical in the pharmaceutical industry as medicinal chemists avoid introducing known toxicophores into their compounds. In this case, the validation dataset was synthesised with guidance from the in-house statistical model, in an attempt to minimise hERG active compounds. This results in a dataset where a number of the active compounds are therefore unexpected actives (black swans) for the previous models and expert knowledge.

**Fig. 2:**
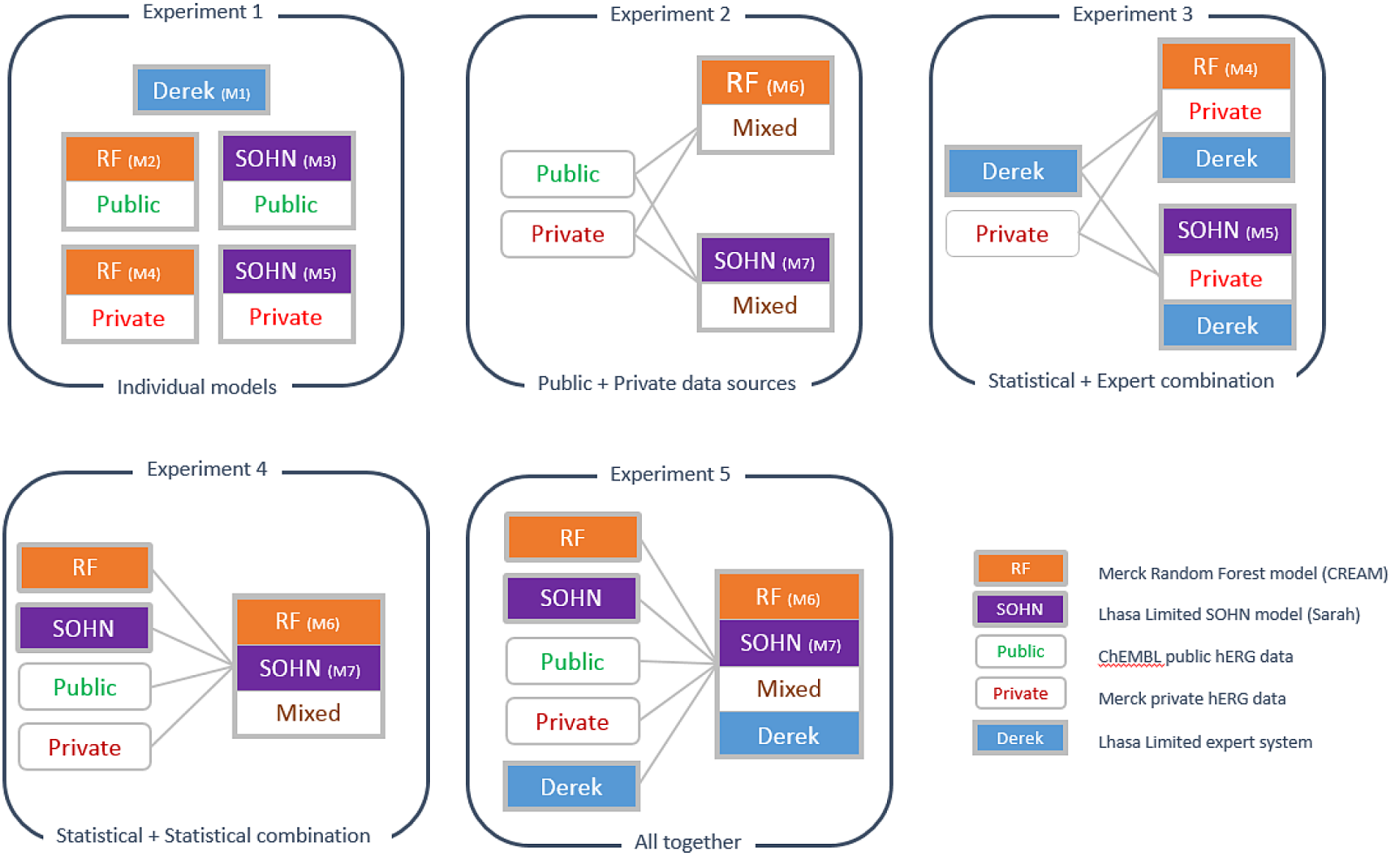
Overview of the evaluation experiments. The evaluation was divided into 5 different experiments addressing different ways of combining the prediction models and the training data sources

Our experimental setup (Fig. 2) was designed to examine the impact that different data sources have on the performance, as well as testing each model individually and determining if they are able to supplement each other to deliver better performance when used in concert.

### Experiment 1: Individual model and individual data sources

The first experiment was designed to check that the tools we are investigating are fit for purpose. It also acted as a baseline to the other experiments. In the experiment, we hope to show that the three different models are appropriate for the prediction of hERG inhibition and also investigate the difference in predictivity in using public versus private data to train the individual statistical models. It was also the first attempt at using the SOHN methodology to predict a receptor based endpoint rather than a reactivity based endpoint and as such it was good to compare it to the RF model.

#### Derek expert system

The Derek expert system relies on a manually compiled knowledge base of structural rules; it is not trained per se with public nor private data and was used as a base line. The rules were developed by a human expert using existing knowledge, literature or looking for SAR trends in both public and donated data. The 316 test compounds were processed in Derek for the mutagenicity endpoint. The results in Table 1 show an accuracy of 75% and an MCC value of 0.27 which indicates a relatively low overall predictivity. The main detrimental factor was the low sensitivity (43%) due to the limited number of hERG alerts present in the current knowledge base and consequently the focused chemical space associated. Derek in isolation isn’t an exhaustive model as it mainly responds to the well-known hERG toxicophores; however we will see later how this focused knowledge of the expert system can still be of benefit when combined with the statistical models.

**Table 1:** Derek Nexus performance against Merck test data

Expert model	ACC	BA	SENS	SPEC	PPV	NPV	MCC	KAPPA
Derek Nexus	0.75	0.63	0.43	0.84	0.44	0.84	0.27	0.27

Although the relatively poor sensitivity of 43% was indicative of the reduced scope of the current knowledge base, Derek’s precision of 44% is considered as a good performance given the strong negative bias of the test dataset; we will see later that Derek is actually outperforming the statistical models trained with public data only in terms of positive predictivity.

#### RF and SOHN statistical models trained with public data

The two statistical models trained only with public data were performing comparably to the Derek expert system (Table 2). The SOHN model displayed a slightly better performance with a relatively good sensitivity whilst keeping a precision (PPV) close to Derek’s. The relatively poor predictivity of the statistical systems can be mostly explained by the difference of chemical space between the private corporate test set (Merck space) and the public data (ChEMBL space). Furthermore, the data collected from ChEMBL came from different primary sources involving a wide range of different protocols. The diversity of assays and protocols in ChEMBL introduced a heterogeneous set of hERG assessments and therefore induced noise in the data due to a lack of normalisation across the different sources.

**Table 2:** RF and SOHN trained with ChEMBL data against the Merck test data

Statistical models (public)	ACC	BA	SENS	SPEC	PPV	NPV	MCC	KAPPA
RF_ChEMBL_	0.74	0.57	0.26	0.88	0.37	0.81	0.16	0.15
SOHN_ChEMBL_	0.73	0.66	0.54	0.78	0.42	0.86	0.30	0.29

It is worth noting that, in regard to positive predictivity, the expert system Derek outperforms the two statistical models; the main reason was the precise structural scope of the hand crafted alerts. Precision is indeed one of the strengths of expert systems and we will see later how this characteristic can be leveraged when combining different approaches.

#### RF and SOHN statistical models trained with private data

When trained with good quality and homogeneous data derived from a single Merck protocol, the statistical models (RF, SOHN) performed much better as shown in Table 3. Both models reached an acceptable predictivity level with a balanced accuracy of 73% and 75% and an MCC value of 0.48 and 0.49 for the RF and SOHN methodologies respectively. Compared to the same models trained with public data, this represents a significant increase in predictivity of about 20%. As it is often the case, compared to public data, private data is usually more homogeneous and naturally closer to the intended application space which results in all performance metrics being significantly enhanced. The results demonstrate the importance of domain proximity and therefore the pivotal role of proprietary data in private corporate domains. This experiment highlights the potential to enhance publicly available models if the knowledge contained in private datasets could be extracted and transferred into such models. Unfortunately, it is very challenging to build public models based on private corporate datasets without leaking information about the highly confidential structures these dataset contain. Different approaches have been considered to overcome this difficulty and it is still an active field [38].

**Table 3:** RF and SOHN trained with Merck data, performance against the Merck test data

Statistical models (private)	ACC	BA	SENS	SPEC	PPV	NPV	MCC	KAPPA
RF_Merck_	0.82	0.73	0.57	0.89	0.61	0.88	0.48	0.47
SOHN_Merck_	0.82	0.75	0.63	0.87	0.59	0.89	0.49	0.48

**Fig. 3:**
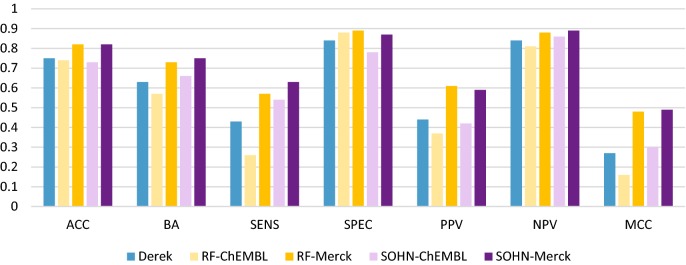
Individual models (Derek Nexus, RF and SOHN) using respectively public and private data. The positive impact in performance of using private data versus public data appears clearly in these results. The expert system Derek Nexus is used as a baseline

We were able to use the performance of the individual models and individual dataset (summarized in Fig. 3) as a baseline to compare the combination of the models and data sources.

### Experiment 2: Combining data sources

**Fig. 4:**
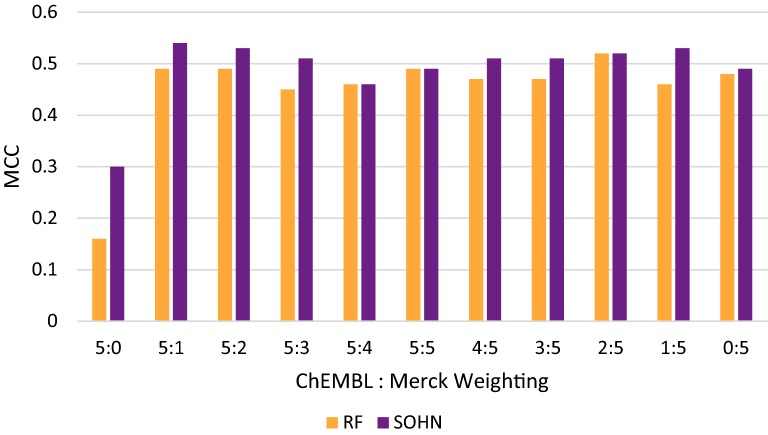
Combining public and private data with different weights

A key question we wanted to investigate was if the combination of public data and private data would result in a better model [39], or if the addition would confuse the models by introducing data from a different area of chemical space. When using the RF methodology and combining the public data with the proprietary Merck data with different weighting factors (sampling ratio), we can see that all weighting schema are performing much better than the public data only and that some schema (2:5, 5:5, 5:1, 5:2) are performing better than both the public data and the private data on their own (Table 4). For instance, the combination of data using the 2:5 ratio for public and private data respectively lead to a very interesting model with a balanced accuracy value up to BA = 75% and an MCC value of 0.52. The same effect was seen with the SOHN methodology. By combining public and private data using different weights we observed better performance than with public data only. This was seen regardless of the ratio used (Table 5). We observed up to 78% for the balanced accuracy and an MCC value of 0.53 with a ratio of 5:1. Although many combinations of data sources demonstrated positive impact on performance, there was no clear pattern in the weighting-schema/performance relationship for both model types (Fig. 4). The access to both chemical spaces enables the RF and the SOHN algorithms to absorb the knowledge present in each domain, independently of the weighing factor. The exact nature of the impact was algorithm dependent and a thorough investigation would require a separate study. While the different public/private combinations performed much better than models trained on public data only (regardless of the model or the public/private ratio), the gain relative to purely private models was less significant due to the already good performance of these models and the expected small domain overlap between the private test space and the public data.

**Table 4:** Combining public and private data for the RF model

Public + private (RF)	ACC	BA	SENS	SPEC	PPV	NPV	MCC	KAPPA
5:0 (100% public)	0.74	0.57	0.26	0.88	0.38	0.81	0.16	0.15
5:1	0.83	0.73	0.56	0.91	0.63	0.88	0.49	0.49
5:2	0.83	0.73	0.56	0.91	0.63	0.88	0.49	0.49
5:3	0.82	0.72	0.53	0.90	0.61	0.87	0.45	0.45
5:4	0.82	0.72	0.53	0.91	0.62	0.87	0.46	0.47
5:5	0.83	0.73	0.56	0.91	0.64	0.88	0.49	0.49
4:5	0.82	0.72	0.54	0.90	0.61	0.87	0.47	0.46
3:5	0.83	0.73	0.54	0.91	0.62	0.88	0.47	0.47
2:5	0.84	0.75	0.59	0.91	0.65	0.89	0.52	0.51
1:5	0.82	0.71	0.51	0.91	0.62	0.87	0.46	0.45
0:5 (100% private)	0.82	0.73	0.57	0.89	0.61	0.88	0.48	0.47

**Table 5:** Combining public and private data for the SOHN model

Public + private (SOHN)	ACC	BA	SENS	SPEC	PPV	NPV	MCC	KAPPA
5:0 (100% public)	0.73	0.66	0.54	0.78	0.42	0.86	0.30	0.29
5.1	0.84	0.78	0.67	0.88	0.62	0.90	0.54	0.53
5:2	0.83	0.78	0.69	0.87	0.60	0.91	0.53	0.53
5:3	0.83	0.76	0.64	0.86	0.61	0.90	0.51	0.51
5:4	0.81	0.73	0.59	0.87	0.57	0.88	0.46	0.45
5:5	0.83	0.74	0.59	0.89	0.61	0.88	0.49	0.48
4:5	0.83	0.76	0.64	0.88	0.60	0.90	0.51	0.51
3:5	0.83	0.76	0.64	0.88	0.60	0.90	0.51	0.51
2:5	0.93	0.76	0.63	0.89	0.62	0.89	0.52	0.52
1:5	0.84	0.77	0.64	0.89	0.64	0.90	0.53	0.53
0:5 (100% private)	0.82	0.75	0.63	0.87	0.59	0.89	0.49	0.48

### Experiment 3: Combining statistical models with an expert model

In this experiment, we evaluated the potential benefit of complementing the statistical models with an expert system. For that purpose we used the ability of the statistical models to express their confidence in a prediction; when this confidence dropped below a defined threshold, we used the expert system as a fall-back predictor. The confidence metrics were specific to each statistical model. However both metrics ranged from 0 to 1 and correlate strongly with the observed accuracy of the predictions. Therefore, we will assume, for the sake of simplification, that confidence values can be compared.

**Fig. 5:**
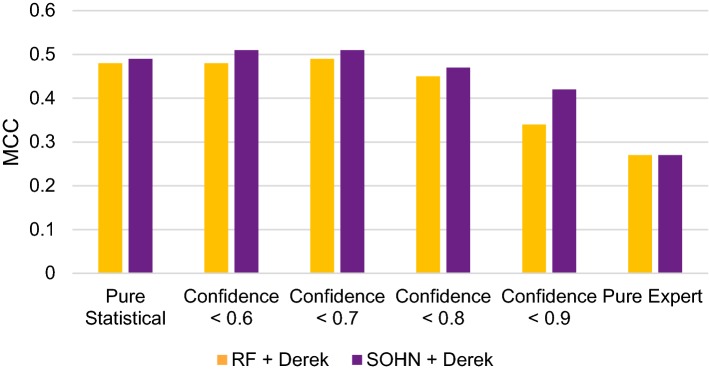
Combining statistical models with the expert model

We compared the performance of the combined statistical-expert systems at different confidence thresholds (Tables 6 and 7, Fig. 5). For both models, the combination with the expert system Derek produced the best performance (based on the MCC) for a confidence threshold value of 0.7. In other words, when the statistical model was less confident (confidence < 0.7) then falling back to the Derek prediction improves the performance. We observe that in both cases (RF and SOHN), the resulting combined model is more sensitive and more precise than the pure expert system. This is the effect of the expert model reducing the number of false positives that occur when the confidence level was low in the statistical models. As mentioned earlier, expert systems like Derek have well defined structural scopes based on existing knowledge; this allows the expert system to exhibit a stronger precision (positive predictivity). The gain in precision observed when using the expert system also means that statistical models, when less confident, tend to make false positive predictions. This is especially apparent when the test space is biased towards negative instances.

**Table 6:** Combining the statistical model RF with the expert system Derek Nexus

RF + Derek Nexus	ACC	BA	SENS	SPEC	PPV	NPV	MCC	KAPPA
Pure statistical (RF)	0.82	0.73	0.57	0.89	0.61	0.88	0.48	0.47
Confidence < 0.6	0.83	0.73	0.56	0.90	0.62	0.88	0.48	0.47
Confidence < 0.7	0.83	0.73	0.54	0.92	0.64	0.88	0.49	0.49
Confidence < 0.8	0.82	0.72	0.53	0.90	0.61	0.87	0.45	0.45
Confidence < 0.9	0.78	0.67	0.47	0.86	0.49	0.85	0.34	0.34
Pure expert (Derek Nexus)	0.75	0.64	0.43	0.80	0.40	0.84	0.27	0.27

**Table 7:** Combining the statistical model SOHN with the expert system Derek Nexus

SOHN + Derek Nexus	ACC	BA	SENS	SPEC	PPV	NPV	MCC	KAPPA
Pure statistical (SOHN)	0.82	0.75	0.63	0.87	0.59	0.89	0.49	0.48
Confidence < 0.6	0.83	0.75	0.60	0.90	0.63	0.89	0.51	0.51
Confidence < 0.7	0.84	0.75	0.59	0.91	0.64	0.89	0.51	0.51
Confidence < 0.8	0.83	0.71	0.49	0.93	0.67	0.86	0.47	0.46
Confidence < 0.9	0.81	0.69	0.47	0.91	0.60	0.86	0.42	0.41
Pure expert (Derek Nexus)	0.75	0.64	0.43	0.80	0.40	0.84	0.27	0.27

In the case of the RF/Derek combination, the MCC value increased from 0.48/0.27 respectively to 0.49 mainly due to an increased precision from 61%/40% to 64% respectively; for the SOHN/Derek combination the MCC is enhanced from 0.49/0.27 to 0.51 when the precision increased from 59%/40% to 64% respectively. We can see that both statistical models benefited from the support of the expert model when they lack confidence. Moreover, the expert system benefited greatly from teaming up with statistical models. These results nicely support the ICH-M7 [40] recommendations of using both expert and statistical systems for predicting mutagenicity. Maintaining and expanding an expert system requires significant effort and domain expertise, making these tasks time consuming. However the resulting models are usually precise. On the other hand, statistical models are easy and fast to build, provided they have access to quality data. Those models are nevertheless not always very precise due to the statistical nature of their algorithm and in regions of low confidence, their predictivity can be questionable. Therefore, both approaches can complement each other into a better meta-model where expert systems act as a “safety net” for statistical models. It is worth noting that not all the combinations in our example lead to positive synergistic effect (i.e. as when the threshold > 0.7). This is a useful reminder that combining an expert model with a statistical model is not guaranteed to produce a better result and in the context of ICH-M7 [40], it raises a clear need for additional expert input.

In this experiment, we saw how the statistical models benefited from the knowledge offered by the expert system when they are not confident. However when the statistical models are confident it is preferable not to use the expert system. By choosing the right confidence threshold, it is possible to build a combination between statistical model and expert model that leverages accuracy of the statistical model when they are confident and the precision of the expert system otherwise.

### Experiment 4: Combining statistical models

**Fig. 6:**
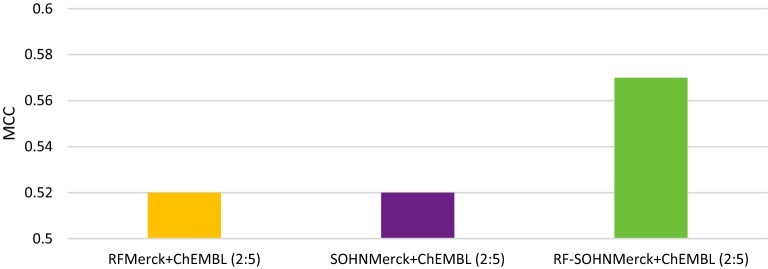
Combining statistical models

The purpose of this experiment was to analyse the impact of combining the predictions of the statistical models together. For that purpose, we used the models (RF and SOHN) trained on combined public and private data, using the same weighting ratio (2:5) to ensure an optimum performance. Thus we should see if their combination is able to further improve the performance. The models were combined by keeping the prediction of the most confident model, i.e. the final call is the call of the model reporting the highest confidence score. The results from Table 8 and Fig. 6 show that the combined model improved both individual models reaching an MCC of 0.57 with a balanced accuracy of 85% and a sensitivity of 66% whilst offering a good precision (67%). This very good overall performance showed the complementarity of the models toward each other thanks to their ability to assign a confidence in their prediction. Estimating the confidence in individual predictions is a challenging task [41], yet it is vital information when combining models.

**Table 8:** Combining statistical models

Models	ACC	BA	SENS	SPEC	PPV	NPV	MCC	KAPPA
RF_Merck+ChEMBL (2:5)_	0.84	0.75	0.59	0.91	0.65	0.89	0.52	0.51
SOHN_Merck+ChEMBL (2:5)_	0.83	0.76	0.63	0.89	0.62	0.89	0.52	0.52
RF-SOHN_Merck+ChEMBL (2:5)_	0.85	0.78	0.66	0.91	0.67	0.90	0.57	0.57

### Experiment 5: Combining all the models and data sources

**Fig. 7:**
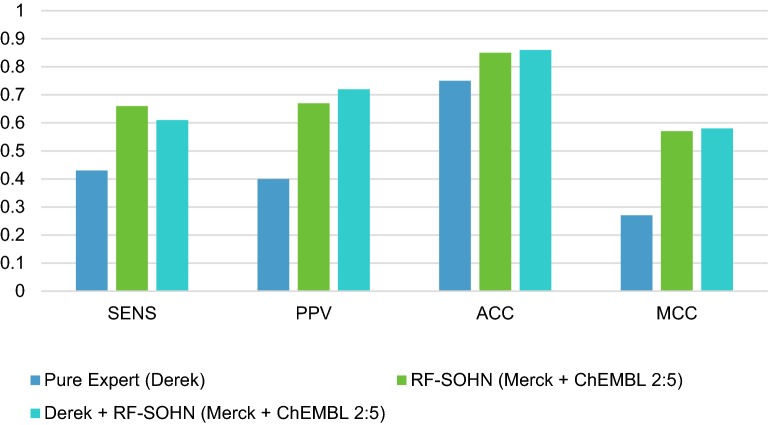
Combining all the models and data sources into a single prediction system. We can observe a light gain in performance mainly driven by the conversion of sensitivity into precision

For the final experiment, we combined all three models to see if we could further extend the synergistic effect. For this purpose, we used the most confident model call as the final call. Both statistical models (RF, SOHN) were trained with a combination of private/public data; we used the best common ratio identified in experiment 2, i.e. 2:5 for public and private data respectively. Given that the Derek Nexus expert system did not provide a normalised confidence metric comparable to the statistical models (Derek uses discrete likelihood range), we used the best confidence threshold when combining Derek with the RF and SOHN models (experiment 3). This threshold of 70% was a logical choice since we have seen that if the statistical models are more confident than 70% we should use their call rather than the Derek call. Table 9 and Fig. 7 show that the overall combination of all three models and both public and private data lead to the best performance. In accordance with experiment 3, the additional use of an expert system on top of statistical models improved the precision or positive predictivity (PPV: + 5%) at the expense of sensitivity (SENS: − 5%) resulting, in this final experiment, with a slight improvement in the Matthew correlation coefficient (MCC: + 0.01) which was our reference metric.

**Table 9:** Combining all the models

Models	ACC	BA	SENS	SPEC	PPV	NPV	MCC	KAPPA
Pure expert (Derek)	0.75	0.64	0.43	0.8	0.4	0.84	0.27	0.27
RF-SOHN_Merck+ChEMBL (2:5)_	0.85	0.78	0.66	0.91	0.67	0.9	0.57	0.57
RF-SOHN_Merck+ChEMBL+Derek (2:5)_	0.86	0.77	0.61	0.93	0.72	0.89	0.58	0.57

## Conclusion

In this work, we have explored different combinations of statistical and expert models with private and public data sources. Individually, each model performed significantly better when trained with private data as opposed to trained with public data. This can be explained by the lack of homogeneity from the public source being compiled from many different assays and quality standards. Another important benefit of private data was the proximity of the chemical space between the private data and the target domain. When the models were trained using both public and private data [39], they usually showed an additional improvement compared to either data. We would therefore recommend combining public and private data whenever possible.

Combining statistical models with the expert model Derek, lead to a good consensus model, if the expert model was used when the statistical model was not confident. The resulting combination outperformed either system on its own. The positive synergistic effect observed was mainly due to a reduction of the false positive predictions, since the expert system tended to be more consistently precise. This result reinforces the ICH-M7 recommendation to use statistical and expert prediction systems in conjunction. When combining statistical systems, using the prediction of the most confident model, we observed another positive synergistic effect. This can be explained by the ability of the two predictors to mutually compensate each other in case of low confidence. When one model is not confident, the other model might be more assertive and vice versa. Both models therefore complemented each other, increasing the overall domain of high confidence and the associated performance.

Finally, we showed in the last experiment that combining all the above models and data sources further increased the performance. The different positive effects from the different nature of the models and the data source were additive. This study allowed us to evaluate the SOHN methodology in the context of a classification task for the hERG endpoint; we demonstrated that the performance of this approach is comparable to the well-established RF algorithm when challenged with a prospective exercise. The SOHN methodology is successfully used to predict mutagenic compounds in the form of the Sarah Nexus software. The good predictivity observed in this study indicates the SOHN to be a promising candidate for binding driven endpoints.

The best model built in this study, by combining the RF and SOHN models both trained with public and private data and backed-up with the Derek expert system, reached a very good accuracy (86%) with a high MCC value (0.58). This performance clearly demonstrates the potential for in silico predictions to improve safety assessment of drugs and reduce the risk of late stage failure in the drug development process. As we saw, one important contribution to this performance is the inclusion of corporate data in the training set. Hence, sharing this valuable “statistical knowledge” across pharmaceutical companies could further improve the models. The main obstacle of such an initiative is the confidentiality of the data, however the huge benefit associated is a strong driver to find new ways to transfer private data into non-confidential models that can be shared across contributors. This is an active field and will be addressed in future collaborative work between Merck KGaA and Lhasa Limited.

## Additional file

.**Additional file 1.** RDKit descriptors used for the Random Forest model.

## Abbreviations

ACC: accuracy
BA: balanced accuracy
hERG: human ether a go go
KAPPA: Cohens kappa coefficient
kNN: *k* nearest neighbours
MCC: Matthews correlation coefficient
NPV: negative predictive value
PPV: positive predictive value
RF: random forest
SENS: sensitivity
SPEC: specificity
SOHN: self organised hypothesis network
TdP: torsade de pointes
