# Supplementary material for: Avoiding hERG-liability in drug design via synergetic combinations of different (Q)SAR methodologies and data sources: a case study in an industrial setting
Source: J Cheminform. 2019 Feb 2;11:9. doi: 10.1186/s13321-019-0334-y (PMC6689868; doi:10.1186/s13321-019-0334-y)
Supplement: Supplementary file 1 — .Additional file 1. RDKit descriptors used for the Random Forest model. [file 13321_2019_334_MOESM1_ESM.docx]

**Additional File 1**

**Metrics**

A comprehensive set of statistical metrics was used for the evaluation of the above described binary classification modelling endeavour. These metrics, which are based on a confusion matrix as described in Table 1, are accuracy (ACC), sensitivity (SENS) (also known as recall or true positive rate), specificity (SPEC) (also known as true negative rate), positive predictive value (PPV) (also known as precision) and negative predictive value (NPV)^27,28^. Furthermore balanced accuracy (BA)^29^, Matthews correlation coefficient (MCC)^30^ and Cohen's kappa coefficient (KAPPA)^31^ were chosen as additional metrics due to their capability of dealing with unbalanced data. All equations adapted for binary classification models are shown below (Eq. 1 -8).

**Table 1**: Confusion matrix for binary classification

|  |  | Predicted Class | |
| --- | --- | --- | --- |
|  |  | active | inactive |
| Experimental  Class | active | **TP** | **FN** |
|  | inactive | **FP** | **TN** |

$\text{ACC}=\frac{TP+TN}{TP+FN+FP+TN}$ (Eq. 1)

$BA=\frac{\frac{TP}{TP+FN}+\frac{TN}{FP+TN}}{2}$ (Eq. 2)

$\text{SENS}=\frac{TP}{TP+FN}$ (Eq. 3)

$\text{SPEC}=\frac{TN}{FP+TN}$ (Eq. 4)

$\text{PPV}=\frac{TP}{TP+FP}$ (Eq. 5)

$\text{NPV}=\frac{TN}{TN+FN}$ (Eq. 6)

$\text{MCC}=\frac{TP\times TN-FP\times FN}{\sqrt{\left( TP+FP \right)\left( TP+FN \right)\left( TN+FP \right)\left( TN+FN \right)}}$ (Eq. 7)

$\text{KAPPA}=\frac{\frac{TP+TN}{TP+FN+FP+TN}-\frac{\left( TP+FP \right)\times\left( TP+FN \right)+(FN+TN)\times(FP+TN)}{{(TP+FN+FP+TN)}^{2}}}{1-\frac{\left( TP+FP \right)\times\left( TP+FN \right)+(FN+TN)\times(FP+TN)}{{(TP+FN+FP+TN)}^{2}}}$ (Eq. 8)

**Random Forest parameters (Python/ScikitLearn)**

from sklearn.ensemble import RandomForestClassifier as Classifier

clf = Classifier(n_estimators=1000, criterion='gini',

max_depth=None, min_samples_split=2, min_samples_leaf=1,

min_weight_fraction_leaf=0.0, max_features='sqrt', max_leaf_nodes=None,

class_weight=None, random_state=rng, n_jobs=16)

**Physico-chemical descriptors used within machine learning**

(calculated with RDKit 2015.09.2)^25^:

- MolWt
- MolLogP
- MolMR
- NumHAcceptors
- NumHDonors
- HeavyAtomCount
- FractionCSP3
- NumAliphaticCarbocycles
- NumAliphaticHeterocycles
- NumAliphaticRings
- NumAromaticCarbocycles
- NumAromaticHeterocycles
- NumAromaticRings
- NumHeteroatoms
- NumRotatableBonds
- TPSA
- PEOE_VSAs
- SMR_VSAs
- SlogP_VSAs
